# Improvement of Outcome for Treatment of ‘Restenosis-prone’ Vascular Lesions? Potential Impact of the Paclitaxel dose on Late Lumen Loss in Porcine Peripheral Arteries

**DOI:** 10.1007/s00270-022-03277-x

**Published:** 2022-09-15

**Authors:** Ole Gemeinhardt, Tobias Haase, Beatrix Schnorr, Jing Xie, Melanie Löchel, Denise Schütt, Antje Mittag, Wolfram Haider, Stephanie Bettink, Ulrich Speck, Gunnar Tepe

**Affiliations:** 1 Department of Radiology, Charité - Universitätsmedizin Berlin, 10117 Berlin, Germany; 2 InnoRa GmbH, Berlin, Germany; 3 Institute of Medical Technology and Research, Rottmersleben, Germany; 4 Institut Für Tierpathologie, Berlin, Germany; 5 Clinical and Experimental Interventional Cardiology, University of Saarland, HomburgSaar, Germany; 6 Department of Radiology, RoMed Klinikum Rosenheim, Rosenheim, Germany

**Keywords:** Balloon angioplasty, Drug-coated balloon, Paclitaxel, Peripheral artery disease, Revascularization, Porcine in-stent stenosis model

## Abstract

**Purpose:**

Clinical data indicate that the drug density on drug-coated balloons (DCBs) might have a role on treatment effect and durability. The aim of the current study was to investigate inhibition of neointimal formation and potential adverse effects after treatment with a novel double-dose DCB in swine.

**Material and methods:**

A four-week study was performed in peripheral arteries of 12 domestic pigs after vessel injury and stent implantation. The novel double-dose DCB with 6-µg paclitaxel (Ptx)/mm^2^ balloon surface (1 × 6) was compared to a standard DCB with 3.5 µg Ptx/mm^2^ (3.5) and uncoated balloons (POBA). Potential adverse effects were stimulated by using three fully overlapping DCBs with 6 µg Ptx/mm^2^ each (3 × 6). Quantitative angiography, histomorphometry and histopathological analyses were performed.

**Results:**

Higher paclitaxel doses per square millimeter of treated arteries were associated with reduced late lumen loss (LLL) in quantitative angiography 4 weeks after treatment (POBA: 0.91 ± 0.75 mm; 3.5: 0.45 ± 0.53 mm; 1 × 6: 0.21 ± 0.41 mm; 3 × 6: − 0.38 ± 0.65 mm). In histomorphometry, maximal neointimal thickness and neointimal area were the lowest for the 1 × 6 group (0.15 ± 0.06 mm/1.5 ± 0.4 mm^2^), followed by 3 × 6 (0.20 ± 0.07 mm/1.8 ± 0.4 mm^2^), 3.5 (0.22 ± 0.12 mm/2.2 ± 1.1 mm^2^) and POBA (0.30 ± 0.07 mm/3.2 ± 0.7 mm^2^). Downstream tissue showed histopathological changes in all groups including POBA, in larger number and different quality (e.g., edema, inflammation, vessel wall necrosis, vasculitis and perivasculitis) in the 3 × 6 group, which did not cause clinical or functional abnormalities throughout the study.

**Conclusion:**

Treatment with the double-dose DCB (6 µg Ptx/mm^2^) tended to increase inhibition of in-stent neointimal formation and to diminish LLL after peripheral intervention in the porcine model compared to a market-approved DCB with 3.5 µg Ptx/mm^2^.

**Supplementary Information:**

The online version contains supplementary material available at 10.1007/s00270-022-03277-x.

## Introduction

In patients with peripheral arterial disease (PAD), angioplasty with drug-coated balloons (DCBs) is an effective treatment associated with high procedural success and prolonged patency compared to plain old balloon angioplasty (POBA). Clinical trials consistently show that DCB treatment reduces late lumen loss (LLL), binary restenosis, and target lesion revascularization (TLR) compared with POBA [1–8]. However, despite the progress that has been made in endovascular treatment, restenosis remains a major issue [9, 10]. One reason for insufficient inhibition of restenosis and long-term patency might be inefficient drug transfer to the vessel wall and/or retention of the drug [11–13].

Several factors that influence local drug delivery and inhibition of neointimal formation have been discussed (e.g., vessel preparation, composition of the coating formulation, excipients, balloon membrane, coating method, balloon-to-vessel diameter ratio, and contact time of the DCB with the vessel wall) [13–15]. Another important factor might be the drug density on the balloon [3, 16]. A drug concentration of 3.0 to 3.5 µg paclitaxel (Ptx)/mm^2^ was proposed as standard dose [17, 18]. Meta-analyses of various studies using paclitaxel-coated balloons (PCBs) for treatment of PAD suggest that low-dose PCBs with 2-µg/mm^2^ balloon surface are associated with premature restenosis and shorter lasting time to reintervention, favoring PCBs with a higher dose of 3.0–3.5 µg/mm^2^ [2, 16, 19, 20]. However, even after treatment with 3.5 µg/mm^2^, treatments in which the effect of therapy does not last long are repeatedly observed [21, 22]. In the COPA CABANA trial, in 22 patients with repeated femoropopliteal in-stent restenosis, treatment with a double dose of Ptx (two fully overlapping DCBs with 3 µg/mm^2^ each) resulted in a smaller LLL after 6 months (0.11 ± 0.78 mm) compared to single-dose treatment (0.34 ± 1.12 mm) or POBA (1.58 ± 1.00 mm) [23]. In the double-dose group, no further TLR was necessary versus 52% in the single-dose group. No adverse events were noticed during the observational period of 20.6 ± 9.4 months. These results suggest that therapeutic outcome may be improved when PCBs with a higher drug density than 3.0–3.5 µg/mm^2^ are used. Until now, a systematic dose-finding study for the treatment of PAD patients with DCBs has not been performed.

In a preliminary study, we have shown that a novel double-dose DCB with 6 µg Ptx/mm^2^ balloon surface enables to deliver at least the same amount of drug as two fully overlapping DCBs with 3 µg Ptx/mm^2^ [14]. In the present study, our aim was to investigate inhibition of neointimal formation and potential adverse effects after treatment with the novel double-dose DCB in the porcine model of peripheral stent- and overstretch-induced neointimal proliferation. The double-dose DCB with 6 µg Ptx/mm^2^ was compared to a standard DCB with 3.5 µg Ptx/mm^2^ and uncoated balloons (POBA). A sixfold standard dose (3 fully overlapping DCBs with 6 µg Ptx/mm^2^ each) was used to provoke adverse effects.

## Materials and Methods

### Study Devices

Conventional angioplasty balloon catheters (135 cm, OTW, wire lumen 0.035″, eucatech AG, Weil am Rhein, Germany) were coated with 6 µg paclitaxel/mm^2^ balloon surface (nominal) in an adjusted formulation with the non-ionic contrast medium iopromide as the major constituent. These catheters were used for treatment with the double-dose DCB (group 1 × 6) and the three fully overlapping DCBs (group 3 × 6). Unused balloons were taken for analysis of total paclitaxel amount on balloon. Drug loss from the DCBs with 6 µg/mm^2^ during advancement to the artery was simulated in vitro (see Appendix A1) [24].

A market-approved PCB with 3.5 µg/mm^2^ (In.Pact Admiral, Medtronic, Minneapolis, MN, USA) was used for single-dose treatment (group 3.5). The PTA balloons of the stent system (Express Vascular SD, Boston Scientific, Maple Grove, MN, USA) were used for uncoated balloon treatment (POBA).

### In vivo Study

The in vivo study (inhibition of neointimal formation after stent implantation and potential adverse effects) was performed in 12 domestic male pigs (body weight 26.4 ± 1.6 kg). The animal experiments were approved by the local animal ethics committee (Saxony-Anhalt, Germany) and were conducted in accordance with European commission directive 86/609/EEC and the German Animal Protection Act.

All animals were treated according to a standard protocol for anesthesia and interventional procedure similar to previous studies (see Appendix A2) [14, 24]. Throughout the interventions, electrocardiogram (ECG) and blood pressure were monitored continuously. Before treatment and at 4-week follow-up, blood samples were taken for complete blood count, and left ventricular ejection fraction (LVEF) was determined (see Appendix A3).

Bare metal stents were implanted in left and right internal iliac and femoral arteries of each animal to enhance neointimal proliferation (4 stents/animal). Afterwards, the stented arterial segments were treated with either one uncoated balloon (POBA) or a standard-dose or double-dose DCB (3.5 or 1 × 6) or three fully overlapping DCBs (3 × 6), achieving a vessel overstretch of about 20%. The four peripheral arteries of each animal were treated with balloons of the same treatment group and the same drug load or uncoated devices, the latter as reference treatment. In each treatment group, three animals were treated. DCB sizes were 5.0 mm in diameter and 40 mm in length for internal iliac and 6.0 mm in diameter and 40 mm in length for femoral arteries. After treatment, all DCBs were collected for residual drug extraction and quantification. Control angiography was performed 4 weeks after treatment. Inhibition of lumen narrowing and neointimal formation versus uncoated balloons were assumed for efficacy.

### Histomorphometry and Histopathological Examination

Stented segments of the treated arteries were dissected for histological examination (see Appendix A4). Injury scores were assigned as previously described by Schwartz et al. [25], and inflammation score was assigned for each stent strut as proposed by Kornowski et al. [26].

Histopathological examination of downstream tissue of muscles and coronary band of the hind limbs were taken. Tissue samples were evaluated by an experienced veterinary pathologist blinded to treatment groups for edema, hemorrhage, degeneration, necrosis, inflammation, scarring, vasculitis/perivasculitis and thrombosis indicating potential damage due microembolism. A total of 11 sites per limb were examined (see Appendix A4).

### Quantitative Angiography (QA)

Offline QA was performed in angiograms taken before, during, and directly after treatment and at 4-week follow-up. Analysis was performed using the software QAngio XA, (Medis, Leiden, the Netherlands).

### Quantification of Paclitaxel on Unused and Used Balloons

Unused and used DCBs were placed in cryovials. Balloons were extracted and paclitaxel content was quantified by high-pressure liquid chromatography (HPLC) with UV detection (Shimadzu Nexera-i LC-2040C 3D, Shimadzu Corporation, Kyoto, Japan) as described before [14].

### Statistical Analysis

For quantitative and qualitative analysis of angiograms, histology, and safety data, observers were blinded to treatment groups. Data are given as mean ± standard deviation. Mean values were calculated for all treated arteries per group. Continuous variables were tested for normal distribution. Comparisons were done either by one-way ANOVA analysis with post hoc Tukey’s test or, if data were not normally distributed, by the Kruskal–Wallis test with Dunn’s post hoc analysis using GraphPad Prism (Version 6, GraphPad Software, San Diego, CA, USA). Since this is an exploratory study, the p-values are descriptive only.

## Results

### Paclitaxel on Balloon Catheters and Used Balloons

Mean paclitaxel doses on balloon used in the 1 × 6 and 3 × 6 groups were 5.5 ± 0.3 µg/mm^2^ (5.0–40 mm, *n* = 4) and 5.6 ± 0.1 µg/mm^2^ (6.0-40 mm, *n* = 4), respectively. Drug loss from these DCBs during in vitro simulation of advancement to the artery was 17.2 ± 3.4% (balloon size 5.0–40 mm, *n* = 3). The proportion of residual drug on the balloon after treatment was similar for all groups (3.5 (*n* = 12): 14.0 ± 5.4%, 1 × 6 (*n* = 12): 13.3 ± 12.3%, 3 × 6 (*n* = 36): 15.3 ± 13.9%.

### In vivo Study

All balloons were successfully deployed. Balloon-to-artery ratios (overstretch) were similar for all treatment groups (*n* = 48 balloons/arteries; 1.19 ± 0.08; p = 0.835; Table 1). Final inflation pressure was 11 ± 2 atm (*n* = 48). The three animals of the 3 × 6 group were treated with the highest drug load. In each of these animals, four arteries were treated with a total of 12 DCBs with 6 µg Ptx/mm^2^ resulting in a total drug load of 59 mg paclitaxel per animal.

**Table 1:** Results of quantitative angiography in internal iliac and femoral arteries

Quantitative angiography					
Group	POBA	3.5	1 × 6	3 × 6	*p*-value (all groups)
n (analyzed vessels)	12	12	12	12	
RFD initial [mm]	4.84 ± 1.07	4.62 ± 0.92	4.34 ± 1.11	4.59 ± 0.80	0.683
MLD in-stent after treatment [mm]	5.26 ± 0.96	5.02 ± 0.82	5.12 ± 0.95	4.96 ± 0.43	0.815
Overstretch [-]	1.18 ± 0.08	1.19 ± 0.10	1.21 ± 0.04	1.19 ± 0.07	0.835
RFD at FU [mm]	4.78 ± 1.08	4.63 ± 0.98	4.40 ± 0.92	4.55 ± 1.01	0.816
MLD in-stent at FU [mm]	4.36 ± 0.97	4.57 ± 0.67	4.92 ± 0.88	5.34 ± 0.88	0.039
LLL in-stent [mm]	0.91 ± 0.75	0.45 ± 0.53	0.21 ± 0.41	− 0.38 ± 0.65	< 0.001
Diameter stenosis in-stent [%]	16.83 ± 15.78	8.28 ± 9.71	3.57 ± 7.84	− 7.48 ± 13.01	< 0.001

Summary of quantitative angiography. RFD initial (vessel reference diameter before first intervention in stented area) [mm], MLD in-stent after treatment (minimal lumen diameter in-stent after implantation and treatment with uncoated or coated balloons) [mm], overstretch (vessel overstretch after stent implantation and treatment with uncoated or coated balloons) [-], RFD at FU (vessel reference diameter at 4-week follow-up) [mm], MLD in-stent at FU (minimal lumen diameter at 4-week follow-up) [mm], LLL in-stent (late lumen loss in-stent) [mm]

No animal died during the study until euthanasia. None of the animals showed clinically abnormal behavior (e.g., reduced feed intake, lameness of hind limbs) or signs of pain during the observational period. Angiography revealed no target-site or downstream thrombi, no thrombemboli and no aneurysms in any of the animals. Four dissections occurred during treatment proximal and/or distal to the stent/balloon in four internal iliac arteries of three animals (one animal of group 3.5 and two animals of group 1 × 6). There was no downflow impairment of flow after treatment in all investigated arteries. For all DCB treatment groups, no obvious deviations to POBA-treated animals were observed for weight gain, blood pressure, ECG, blood parameters (Appendix Table B1), and LVEF (Table 2).

**Table 2:** Weight gain of the animals, difference in blood pressure during anesthesia and treatment, and left ventricular ejection fraction (LVEF) before and 4 weeks after treatment

Group	Efficacy and tolerance study				
	POBA	3.5	1 × 6	3 × 6	*p*-value (all groups)
n (animals per group)	3	3	3	3	
Weight gain [kg]	3.8 ± 1.3	5.7 ± 2.4	5.5 ± 1.5	4.7 ± 1.0	0.504
Delta blood pressure [mmHg]	− 24 ± 10	− 14 ± 3	− 16 ± 7	− 16 ± 13	0.516
Baseline LVEF	44.4 ± 4.1	41.3 ± 7.7	47.9 ± 5.3	42.0 ± 2.5	0.443
4-week LVEF	40.7 ± 2.3	37.7 ± 6.5	41.3 ± 9.2	41.0 ± 3.3	0.935
Delta LVEF	− 3.7 ± 3.0	− 3.6 ± 2.1	− 6.6 ± 4.8	− 1.0 ± 1.3	0.508

Weight gain of the animals during study period [kg], delta of systolic blood pressure [mmHg] before treatment and after treatment of the last artery of each animal (reduction of blood pressure is mainly due to anesthesia), LVEF: left ventricular ejection fraction [%] before stent implantation and at 4-week follow-up (4-week LVEF), and the difference from baseline to 4-week LVEF (Delta LVEF)

### Quantitative Angiography (QA)

Results of QA (Table 1) showed similar values of the treatment groups for vessel reference diameter in the stented area before stenting (RFD, *p* = 0.683), in-stent minimal lumen diameter (MLD) directly after treatment (*p* = 0.815), and overstretch ratio (*p* = 0.835).

**Fig. 1:**
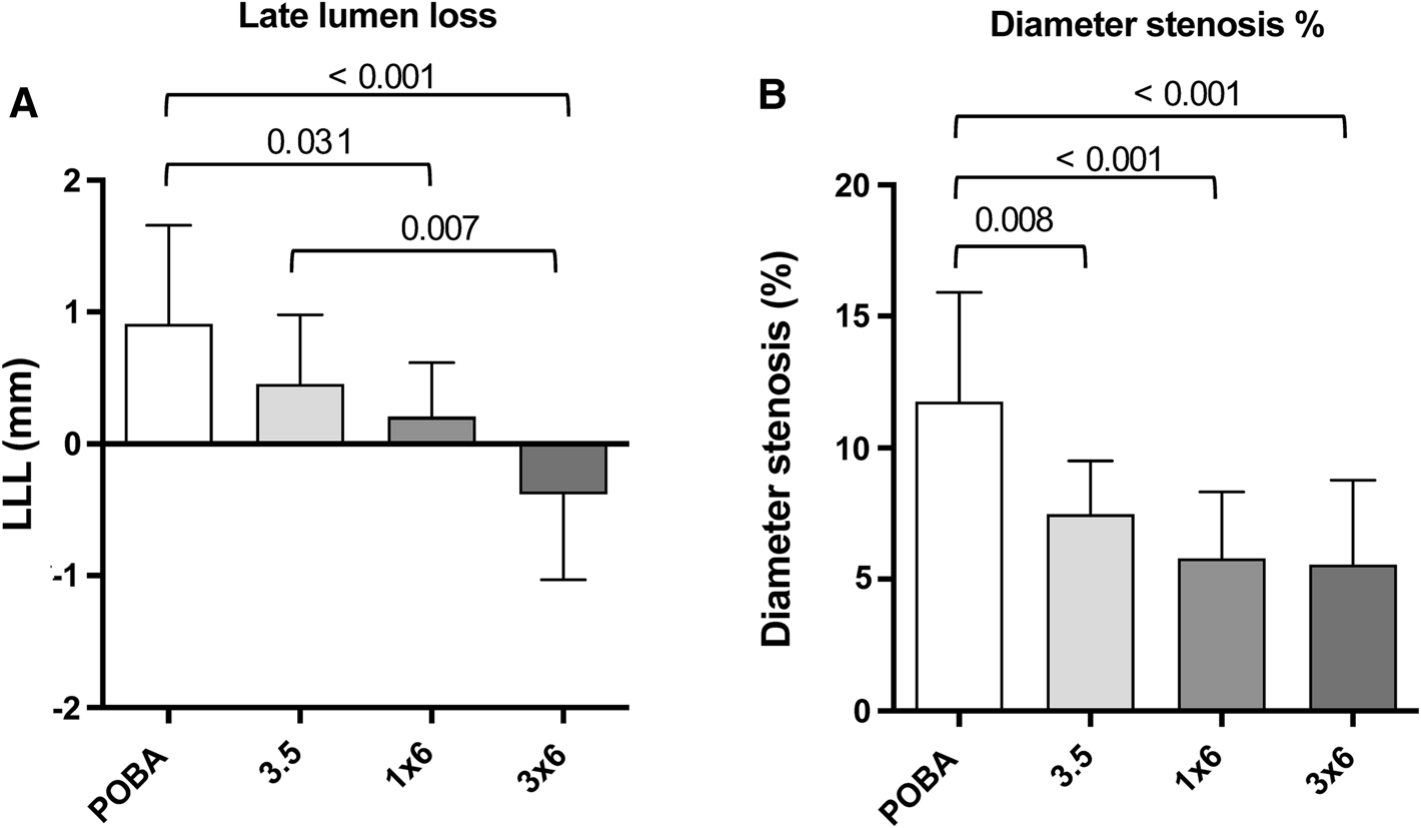
Summary of QA and histomorphometry results: LLL (QA) and diameter stenosis (histomorphometry). (**A**) Quantitative angiography of late lumen loss (LLL) after 4 weeks and (**B**) histomorphometry of diameter stenosis at 4-week follow-up. Differences between treatment groups are shown. Since this is an exploratory study, the *p*-values are descriptive only

Four weeks after treatment, QA revealed a lower LLL with increasing paclitaxel doses per artery (POBA: 0.91 ± 0.75 mm; 3.5: 0.45 ± 0.53 mm; 1 × 6: 0.21 ± 0.41 mm; 3 × 6: − 0.38 ± 0.65 mm; Fig. 1).

When comparing two groups, differences according to post hoc analyses were found for: MLD in-stent at FU = 3 × 6 vs POBA (*p* = 0.035); LLL in-stent = 1 × 6 vs POBA (*p* = 0.031); 3 × 6 vs POBA (*p* < 0.001); 3 × 6 vs 3.5 (*p* = 0.007); diameter stenosis in-stent = 1 × 6 vs POBA (*p* = 0.045); 3 × 6 vs POBA (*p* < 0.001); 3 × 6 vs 3.5 (*p* = 0.012).

### Histomorphometry

Results of histomorphometry are compiled in Table 3. At 4 weeks after treatment, the vessel diameters were similar in all four groups (*p* = 0.798). Neointimal thickness and neointimal area were the smallest in the 1 × 6 group (0.15 ± 0.06 mm/1.5 ± 0.4 mm^2^), followed by the 3 × 6 (0.20 ± 0.07 mm/1.8 ± 0.4 mm^2^) and 3.5 group (0.22 ± 0.12 mm/2.2 ± 1.1 mm^2^). The highest values for neointimal formation were observed in the POBA group (0.30 ± 0.07 mm/3.2 ± 0.7 mm^2^). No differences between the groups were found for overstretch-induced injury score (*p* = 0.301) and inflammation score (*p* = 0.146).

**Table 3:** Results of histological examination of internal iliac and femoral arteries

Histomorphometry					
Group	POBA	3.5	1 × 6	3 × 6	*p*-value (all groups)
n (analyzed vessels)	12	12	12	12	
Vessel diameter [mm]	4.75 ± 0.93	4.58 ± 0.89	4.45 ± 0.87	4.75 ± 0.83	0.798
Maximal neointimal thickness [mm]	0.30 ± 0.07	0.22 ± 0.12	0.15 ± 0.06	0.20 ± 0.07	< 0.001
Neointimal area [mm^2^]	3.17 ± 0.65	2.16 ± 1.14	1.45 ± 0.41	1.82 ± 0.45	< 0.001
Injury score [-]	0.0 ± 0.1	0.1 ± 0.1	0.0 ± 0.0	0.0 ± 0.1	0.301
Artery inflammation score [-]	1.7 ± 0.2	1.8 ± 0.2	1.7 ± 0.2	1.6 ± 0.2	0.146
Diameter stenosis [%]	11.75 ± 4.16	7.47 ± 2.03	5.79 ± 2.54	5.55 ± 3.22	< 0.001
Lumen loss [%]	22.17 ± 7.49	14.04 ± 4.08	11.53 ± 4.56	12.47 ± 4.60	< 0.001

Summary of histomorphometry in-stent 4 weeks after treatment. Vessel diameter [mm], maximal neointimal thickness [mm], neointimal area [mm^2^], injury score [–], artery inflammation score [–], diameter stenosis [%] and lumen loss [%]

When comparing two groups, differences according to post hoc analyses were found for: maximal neointimal thickness = 1 × 6 vs POBA (*p* < 0.001); 3 × 6 vs POBA (*p* = 0.026); neointimal area = 3.5 vs POBA (*p* = 0.007); 1 × 6 vs POBA (*p* < 0.001); 3 × 6 vs POBA (*p* < 0.001); diameter stenosis = 3.5 vs POBA (*p* = 0.008); 1 × 6 vs POBA (*p* < 0.001); 3 × 6 vs POBA (*p* < 0.001); lumen loss = 3.5 vs POBA (*p* = 0.003); 1 × 6 vs POBA (*p* < 0.001); 3 × 6 vs POBA (*p* < 0.001).

### Histopathological Examination of Downstream Tissues

**Fig. 2:**
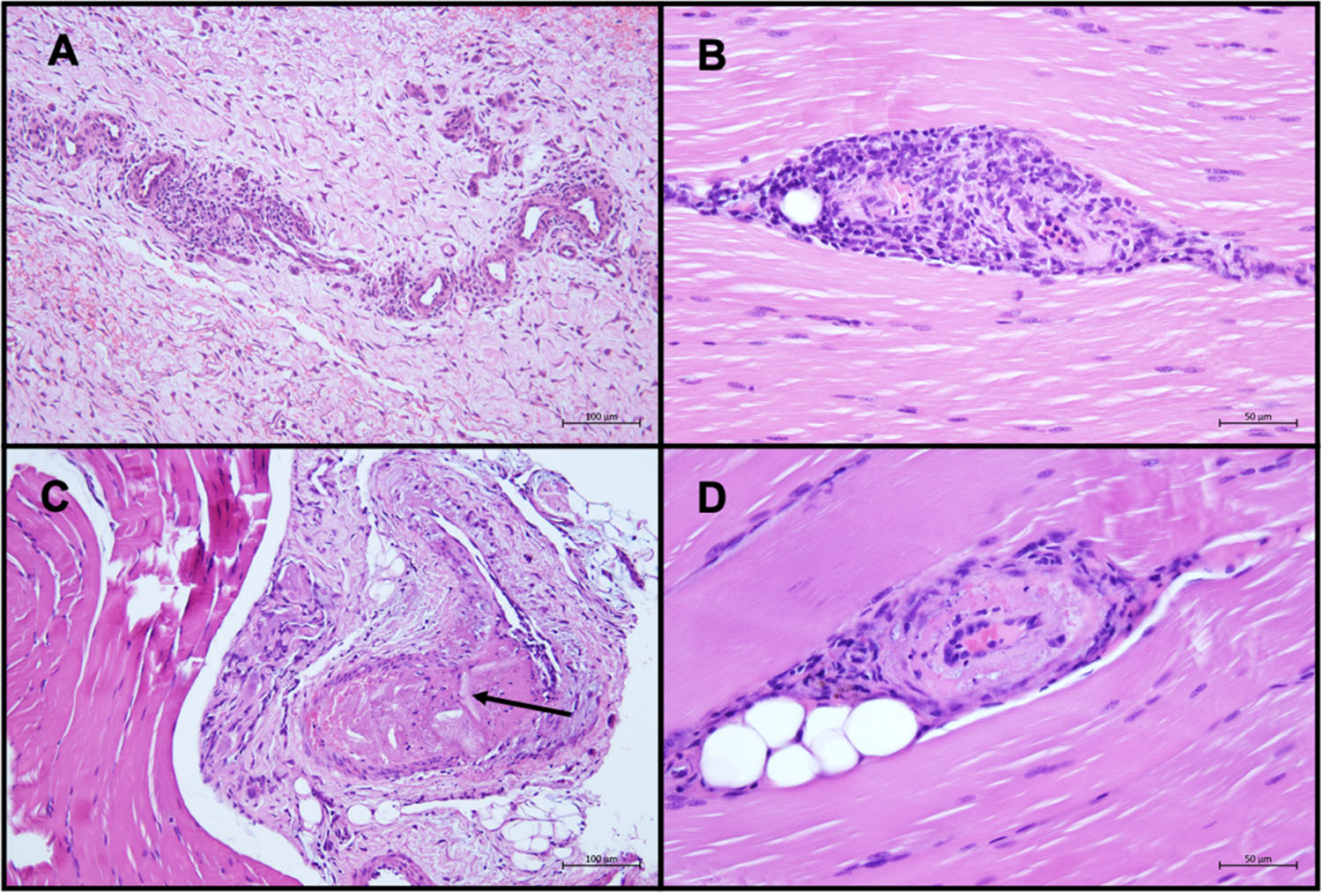
Representative HE-stained histological images of muscle tissue. Representative images of hematoxylin and eosin (HE)-stained sections of muscle tissue showing perivasculitis (**A**), vasculitis and perivasculitis (**B**), thrombus with unidentified material (arrow), but without inflammation (**C**), and necrosis and perivasculitis of the vessel wall (**D**). Magnification 200x or 400x

Lesions were classified as not detected, very low grade, low grade, medium grade and high grade (Table 4). Changes in downstream tissue were seen in all groups including POBA, in larger number and different quality in the 3 × 6 group. A total of five animals (one animal of group 1 × 6, two animals of group 3.5 and 3 × 6 each) showed evidence of downstream effects in the examined muscle tissue sections, likely resulting from DCB treatment (e.g., vessel wall necrosis, vasculitis and perivasculitis; Fig. 2A, B and D). These changes were mostly classified as low-grade changes. One finding out of 66 sites per DCB group each was classified as medium grade (3.5 and 3 × 6 vasculitis; 1 × 6 hemorrhage). None of these changes did cause vascular occlusion or necrosis in the supplied tissue. These changes did not occur in the POBA group, but other changes (e.g., inflammation) were also detected in the POBA group. In one animal (group 3 × 6), the relationship to DCB treatment was not clear, because the affected vessels only showed perivasculitis. In one affected vessel, unidentified material was detected. Thrombosis also occurred here, while inflammation was not present (Fig. 2C).

**Table 4:** Summary of histopathological findings

Diagnosis	Animals (legs) with findings	Gracilis muscle		Gastrocnemius muscle		Ext. digit. brevis muscle		Animals (legs) with findings	Coronary band (dig. 3 + 4)	
		(*n* = 6 sites/group)		(*n* = 6 sites/group)		(*n* = 18 sites/group)			(*n* = 36 sites/group)	
	Number	Number	Severity	Number	Severity	Number	Severity	Number	Number	Severity
**POBA,** **no coating**										
Edema	3 (4)	1	+	2	+ / +	6	+ / + / + / + / + / +	0 (0)	–	–
Hemorrhage	0 (0)	–	–	–	–	–	–	0 (0)	–	–
Degeneration	0 (0)	–	–	–	–	–	–	0 (0)	–	–
Necrosis	0 (0)	–	–	–	–	–	–	0 (0)	–	–
Inflammation	1 (1)	1	+	–	–	–	–	2 (3)	6	+ / ++ / + / + / + / +
Scarring	0 (0)	–	–	–	–	–	–	0 (0)	–	–
Vasculitis	0 (0)	–	–	–	–	–	–	0 (0)	–	–
Thrombosis	0 (0)	–	–	–	–	–	–	0 (0)	–	–
Overall findings in the POBA group: 16 from 66 sites										
**3.5** **µg** **Ptx/mm**^**2**^										
Edema	2 (2)	–	–	–	–	2	+ / +	0 (0)	–	–
Hemorrhage	0 (0)	–	–	–	–	–	–	0 (0)	–	–
Degeneration	0 (0)	–	–	–	–	–	–	0 (0)	–	–
Necrosis	0 (0)	–	–	–	–	–	–	0 (0)	–	–
Inflammation	0 (0)	–	–	–	–	–	–	2 (2)	2	+ / +
Scarring	0 (0)	–	–	–	–	–	–	0 (0)	–	–
Vasculitis	2 (3)	2	+ / ++	1	+	1	+	0 (0)	–	–
Thrombosis	0 (0)	–	–	–	–	–	–	0 (0)	–	–
Overall findings in the 3.5-group: 8 from 66 sites										
**1 × 6** **µg** **Ptx/mm**^**2**^										
Edema	2 (2)	1	+	1	+	3	+ / + / +	0 (0)	–	–
Hemorrhage	1 (1)	–	–	–	–	1	+ +	0 (0)	–	–
Degeneration	0 (0)	–	–	–	–		–	0 (0)	–	–
Necrosis	1 (1)	1	+	–	–	–	–	0 (0)	–	–
Inflammation	1 (1)	–	–	–	–	1	+	1 (2)	2	+ / +
Scarring	0 (0)	–	–	–	–	–	–	0 (0)	–	–
Vasculitis	1 (1)	1	+	–	–	–	–	0 (0)	–	–
Thrombosis	0 (0)	–	–	–	–	–	–	0 (0)	–	–
Overall findings in the 1 × 6-group: 11 from 66 sites										
**3** **×** **6** **µg** **Ptx/mm**^**2**^										
Edema	2 (3)	1	+	–	–	5	+ / + / + / + / +	0 (0)	–	–
Hemorrhage	0 (0)	–	–	–	–	–	–	0 (0)	–	–
Degeneration	0 (0)	–	–	–	–	–	–	0 (0)	–	–
Necrosis	2 (2)	2	+ / +	–	–	–	–	0 (0)	–	–
Inflammation	1 (2)	1	+	–	–	4	+ / + / + / +	2 (2)	5	+ / + / + / + / +
Scarring	0 (0)	–	–	–	–	–	–	0 (0)	–	–
Vasculitis*	3 (4)	4	+ / ++ / + / +	–	–	2	+ / +	1 (1)	2	+ / +
Thrombosis	1 (1)	1	+	–	–	–	–	0 (0)	–	–
Overall findings in the 1 × 6-group: 27 from 66 sites										

Diagnoses of pathological findings, number of findings (sum of all limbs per group) and severity are given

Severity of the changes was rated as follows: − = not detected, ( +) = very low grade, + = low grade, ++ = medium grade and +++ = high grade

^*^ In one animal only perivasculitis (without vasculitis) was detected

In the coronary band tissue, low-grade inflammation and vasculitis were seen, usually localized subepidermally. These changes did not correlate with DCB treatment and were most likely attributable to external causes (e.g., fixation of the legs during anesthesia, trauma), which led to microlesions of the skin in this area. Histopathological changes, related to balloon treatment, were not observed in coronary band tissue.

## Discussion

Clinical observations of the COPA CABANA trial [23] indicate that treatment of patients with PAD with a double-dose paclitaxel may prevent restenosis in in-stent stenosis, even after failure of previous treatments with DCB or drug-eluting stent. In our study, we examined treatment efficacy (e.g., lumen preservation and neointimal formation) and potential adverse effects of a novel double-dose DCB with 6 µg Ptx/mm^2^ balloon surface in the animal model of in-stent stenosis. Peripheral arteries of 12 pigs were treated with uncoated or paclitaxel-coated balloons after induction of neointimal formation by means of stent implantation and vessel overstretch. Treatment with the double-dose DCB (1 × 6) was compared to a standard DCB with 3.5 µg Ptx/mm^2^ (3.5) and uncoated balloons (POBA). A sixfold standard dose (3 fully overlapping DCBs with 6 µg Ptx/mm^2^ each, 3 ×  6) was used to explore potential adverse effects not seen at single balloon application. Treatment with the double-dose DCB showed a tendency to increase inhibition of in-stent neointimal formation and to diminish LLL compared to the already excellent efficacy of the market-approved DCB with 3.5 µg Ptx/mm^2^ in the animal model. Histopathological examination of downstream tissue revealed some tissue changes associated with DCB treatment (e.g., vessel wall necrosis, vasculitis and perivasculitis), which did not cause clinical or functional abnormalities throughout the study.

In one randomized, non-inferiority clinical trial [27], patients with symptomatic femoropopliteal lesions were treated with a DCB either with a drug load of 2 µg Ptx/mm^2^ (Ranger Paclitaxel-Coated PTA Balloon Catheter, Boston Scientific, Marlborough, MA, USA) or 3.5 µg Ptx/mm^2^ (In.Pact Admiral or In.Pact Pacific, Medtronic Vascular, Santa Clara, CA, USA). At 12-month follow-up, results of the two groups were similar for primary patency (2 µg/mm^2^ 83.0%; 3.5 µg/mm^2^ 81.5%) and freedom from major adverse events (2 µg/mm^2^ 91.0%; 3.5 µg/mm^2^ 92.6%). Data for long-term outcome of this study would be of great interest. In contrast, other clinical data indicate that the paclitaxel dose on DCBs is correlated with treatment efficacy in patients with PAD [2, 3, 16]. Lesions with high risk for recurrence such as in-stent restenosis, restenosis after DCB treatment, or patients with diabetes mellitus or renal insufficiency might require a higher drug dose to achieve the sufficient and persistent efficacy [2, 3]. The data of the COPA CABANA study and the results of the reported study in swine suggest a randomized clinical trial to investigate a clinical usefulness of a double-dose treatment.

Besides treatment of vascular stenosis of the lower limb balloon angioplasty has become an important tool in the treatment of stenosed or occluded hemodialysis arteriovenous (AV) fistulas (AV shunt stenosis). Several studies have shown benefits of PCBs in the treatment of AV shunt stenosis resulting in fewer re-interventions and a higher primary patency compared to POBA [22, 28, 29]. However, patency rates over six months still leave room for improvement. Among other things, the observed variability in efficacy and failure to significantly extend long-term patency may indicate insufficient delivery or retention of the drug [29].

In a first preclinical study, we have shown that a single DCB with a double dose of 6 μg Ptx/mm^2^ enables doubling of drug transfer to peripheral arteries compared to a single dose of 3 μg Ptx/mm^2^ [14]. Until now, no dose-finding study for treatment of PAD patients with DCBs has been performed. Kelsch et al. investigated different drug doses using the porcine model of coronary in-stent stenosis [17]. In this study, treatment with 9 µg Ptx/mm^2^ did not improve treatment efficacy compared to 3 µg Ptx/mm^2^ (LLL 28 days after treatment: 9 µg Ptx/mm^2^ 0.19 ± 0.11 mm; 3 µg Ptx/mm^2^ 0.20 ± 0.11 mm; POBA 1.21 ± 0.51 mm). In our present study, treatment with the double-dose DCB with 6 µg Ptx/mm^2^ resulted in less LLL and stenosis in QA and in less neointimal thickness, neointimal area and stenosis in histomorphometry compared to the DCB with 3.5 µg Ptx/mm^2^ and to POBA. A further increase in the drug dose using the three fully overlapping double-dose DCBs did not result in a further increase in the inhibition of neointimal formation.

Technically, all balloons coated with 6 µg Ptx/mm^2^ were successfully deployed. Drug loss from the novel DCB during in vitro simulation of advancement to the artery was acceptable with 17.2 ± 3.4% of the balloon dose (balloon size: 5.0–40 mm). Drug release was similar for all groups with residual amounts of paclitaxel after treatment of 13% to 15% of the balloon dose and similar to the range of 5% to 23% reported by other preclinical studies [13, 14, 30].

In our study, none of the animals showed clinically abnormal behavior or signs of pain during the study period. After PCB treatment, no obvious deviations to POBA-treated animals were observed for weight gain, blood pressure, ECG, blood parameters, and LVEF even after treatment with three fully overlapping DCBs with 6 µg Ptx/mm^2^ per arterial segment and four treated arteries per animal. Potential changes in downstream tissue after DCB treatment have been widely discussed [24, 31, 32]. Changes in downstream tissue were also shown by histopathological examination in the present study in all groups including POBA, in larger number and different quality in the 3 × 6 group. In some cases, small vessel wall necrosis or vasculitis and perivascular inflammation in downstream tissue likely resulting from DCB treatment was shown. However, these changes were classified as low grade, except one medium grade finding out of 66 sites in each DCB group. Only muscle tissue was affected by these changes but not coronary band tissue. These findings confirm the conclusion of several other preclinical studies that none of the changes observed in downstream tissue indicate clinically relevant damage [13, 24, 33].

To what extend the inflation of three fully overlapping balloons of the 3 × 6 group influenced (benefited or harmed) the results of our study, independent of the paclitaxel, were not examined and cannot be derived from this study.

Concerns that local endovascular therapy with paclitaxel-coated devices might be associated with higher mortality rates were first voiced some years ago [34]. In several randomized trials and large bodies of real-world data, these concerns were not confirmed [1, 6, 35–40].

### Limitations of the study

The main limitation of the study derives from the nature of large animal experimental models. In our study, we treated young healthy pigs neglecting typical tissue characteristics of stenotically altered blood vessels in humans. The effects of the treatment were recorded over 4 weeks and not over several years, as is desirable in clinical trials. As usual with large animal models, the number of animals was limited. As standard DCB, we used a market-approved DCB not only with a different drug load of 3.5 µg Ptx/mm^2^, but also with different coating formulation.

The results of our study may indicate a possible benefit of treatment with the double-dose DCB, but the results cannot be extrapolated to any clinical setting. Therefore, only clinical trials using a double dose for treatment can provide information about long-term treatment efficacy and potential safety risks in patients.

## Conclusions

Treatment with a novel double-dose DCB (6 µg Ptx/mm^2^) showed a tendency to improve inhibition of in-stent neointimal formation and to diminish LLL after peripheral intervention in the porcine model of in-stent stenosis compared to a market-approved DCB with 3.5 µg Ptx/mm^2^. Histopathological analyses of downstream tissue revealed mostly low-grade changes attributable to DCB treatment, which did not cause clinical abnormalities during the study period. Treatment with the novel double-dose DCB might has the potential to improve inhibition of neointimal formation. Only clinical trials using the tested DCB versus a standard DCB for treatment can provide information about long-term treatment efficacy and potential safety risks in patients.

## Supplementary Information

Below is the link to the electronic supplementary material.

Supplementary file1 (PDF 230 KB)
