# Supplementary material for: Improvement of Outcome for Treatment of ‘Restenosis-prone’ Vascular Lesions? Potential Impact of the Paclitaxel dose on Late Lumen Loss in Porcine Peripheral Arteries
Source: Cardiovasc Intervent Radiol. 2022 Sep 15;45(12):1822–31. doi: 10.1007/s00270-022-03277-x (PMC9705460; doi:10.1007/s00270-022-03277-x)
Supplement: Supplementary file 1 — Supplementary file1 (PDF 230 KB) [file 270_2022_3277_MOESM1_ESM.pdf]

Appendix A. Methods

A1. In vitro simulation of drug loss during advancement to the artery

Drug-coated PTA balloons (6 µg paclitaxel/mm^2, balloon size 5.0-40 mm, n=3) were passed through a blood-filled introductory sheath (7F, 40 cm, Flexor® Check-Flo® Introducer with membrane valve, Cook Medical, USA) over a 0.035" guide wire. Balloons were advanced in a vial filled with heparinized porcine blood stirred at 37°C. The balloons were kept in blood for 1min, then taken out (without being retracted into the introductory sheath) and collected for residual drug quantification.

A2. Anesthesia and interventional procedure

Dual antiplatelet therapy (clopidogrel, acetylsalicylic acid), prevention of vascular spasm during procedures (long-acting verapamil), sedation (ketamine, xylazine hydrochloride), general anesthesia (propofol, isoflurane), pain medication, and vascular access via the common carotid artery are described elsewhere [1]. Ursocyclin (20 mg/kg, IM) was administered as antibiotic therapy. Daily acetysalicylic acid (100 mg/kg) and clopidogrel (75 mg/kg) were given orally until end of study.

Imaging was performed using a Siemens AXIOM Artis zee fluoroscope. Under fluoroscopic control a guiding catheter was introduced through the arterial sheath over a guidewire and advanced into the aorta. Internal iliac and femoral arteries were visualized using an approved nonionic iodinated contrast agent. For QA, radiographs of native vessels, images with stents, and inflated balloons were taken as well as images documenting final results after treatment of each artery and at 4-week follow-up. Throughout the interventions, electrocardiogram (ECG), SpO_2, and body temperature were monitored continuously.

After baseline angiography one bare metal stent (Express Vascular SD, Boston Scientific, USA) per vessel was implanted in each internal iliac (stent size: 5.0-19 mm) and femoral artery (stent size: 6.0-18 mm), without major overstretch.

Directly after stent implantation, the arteries were treated with either one uncoated (POBA) or one drug-coated balloon (3.5 group or 1x6 group) or three fully overlapping drug-coated balloons (3x6 group) applying appropriate inflation pressure to achieve about 20% overstretch compared with the reference diameter of the vessel. Inflation time was 60 seconds per balloon. Balloon sizes were 5.0-40 mm for internal iliac and 6.0-40 mm for femoral arteries. Each balloon was placed to fully cover the implanted stent. In the group treated with three DCBs (3x6), inflation pressure was increased with each balloon by 1-2 atm. The last balloon of each vessel should reach 20% overstretch. In the POBA group, the PTA balloons of the stent system were used for uncoated balloon treatment. After stent implantation, the uncoated balloon was deflated and inflated again in the same position. Afterwards, each balloon was deflated and retracted. All DCBs were collected for residual drug quantification. Following completion of all study interventions, the carotid arteriotomy was sutured, and the dermal layers closed using standard techniques.

Control angiography was performed 4 weeks after treatment. The animals were treated and anesthetized as described above, except antibiotic therapy. Angiography was performed for offline evaluation of stent deformation and quantitative angiography (QA). After final angiography the animals were euthanized in deep anesthesia using an intravenous bolus of 10ml super-saturated potassium chloride. Directly afterwards samples for histological examination were taken. All tissue samples were rapidly fixed with 10% buffered formalin.

A3. Left ventricular ejection fraction (LVEF)

For determination of the LVEF an iodinated contrast medium (Ultravist 370, Bayer Vital, Germany) was injected in the left ventricle via a 6F pigtail catheter using the Angiomat 3000 (Liebel-Flarsheim Co. LLC, USA). The injection volume was 20ml and injection rate 15 ml/second. Images were acquired at LAO 30°/0° with 30 images per second. Quantification of LVEF was performed at the Artis Zee Axiom Workstation based on contour information in the end-diastolic and end-systolic image fields in combination with pixel size.

LVEF = ratio of the difference of end-diastolic volume (EDV) and end-systolic volume (ESV) to EDV in percent: 100% x (EDV-ESV)EDV.

A4. Histomorphometry and histopathological examination

Stented segments of the treated arteries plus ≥ 1 cm of the vessel proximal and distal to the stent were dissected for histological examination. The dissected arterial segments including the stents were embedded in methyl-methacrylate, sectioned, and stained as described by Kamann et al. [2]. Cross sections of three segments (proximal, mid, distal) within the stented area were digitalized on a slide scanner (Nanozoomer, Hamamatsu, Japan) and analyzed using NDP.view software (Hamamatsu, Japan). Histomorphometric data of the three cross-sectional segments were averaged to obtain a mean value per stent.

For examination of potential downstream effects tissue samples of gracilis, gastrocnemius and extensor digitorum brevis muscles of both hind legs as well as two samples of the coronary band of the third and fourth toes were sampled from each pig. One proximal, one medial, and one distal cross section of each extensor digitorum brevis muscle was taken as well as abaxial, medial and axial cross sections of each coronary band. From the gracilis muscle and gastrocnemius muscle, only one cross section each was taken for investigation. Tissue samples for histopathological examination were fixed in formalin, embedded in paraffin, sectioned, and stained with HE.

Appendix B. Tables

Table B1: Summary of blood parameters before treatment and at 4-week follow-up

Treatment group and No. of pig	Erythrocytes		Hematocrit		Hemoglobin		Leukocytes	
	Pre int.	4-week FU	Pre int.	4-week FU	Pre int.	4-week FU	Pre int.	4-week FU
Standard value for pigs	5.8-8.1		0.33-0.45		108-148		10.0-22.0	
	[T/l]	[T/l]	[l/l]	[l/l]	[g/l]	[g/l]	[G/l]	[G/l]
POBA-1	6.13	6.61	0.31	0.31	97	105	17.9	20.9
POBA-2	5.18	5.90	0.29	0.30	93	105	14.4	10.6
POBA-3	6.85	7.00	0.34	0.33	113	113	15.2	16.4
Mean ± SD	6.05 ± 0.84	6.50 ± 0.56	0.31 ± 0.33	0.31 ± 0.02	101 ± 11	108 ± 5	15.8 ± 1.8	16.0 ± 5.2
3.5-1	5.44	5.69	0.29	0.30	94	96	17.7	13.5
3.5-2	6.01	6.03	0.32	0.30	102	99	18.0	14.2
3.5-3	6.50	5.67	0.31	0.28	102	92	14.2	13.0
Mean ± SD	5.98 ± 0.53	5.80 ± 0.20	0.31 ± 0.02	0.29 ± 0.01	99 ± 5	96 ± 4	16.6 ± 2.1	13.6 ± 0.6
1x6-1	6.22	7.06	0.32	0.34	100	111	14.7	14.9
1x6-2	5.50	6.86	0.32	0.38	102	125	11.6	18.4
1x6-3	5.96	5.70	0.30	0.28	97	93	15.5	16.8
Mean ± SD	5.89 ± 0.36	6.54 ± 0.73	0.31 ± 0.01	0.33 ± 0.05	100 ± 3	110 ± 16	13.9 ± 2.1	16.7 ± 1.8
3x6-1	6.62	6.75	0.34	0.33	108	111	11.7	12.5
3x6-2	5.43	4.73	0.31	0.26	103	87	12.8	12.6
3x6-3	5.84	5.75	0.33	0.31	107	106	11.4	12.6
Mean ± SD	5.96 ± 0.60	5.74 ± 1.01	0.33 ± 0.02	0.30 ± 0.04	106 ± 3	101 ± 13	12.0 ± 0.7	12.6 ± 0.1

Group-Animal	Monocytes		Hypochromia		Anisocytosis		Thrombocytes	
	Pre int.	4-week FU	Pre int.	4-week FU	Pre int.	4-week FU	Pre int.	4-week FU
Standard value for pigs	2-4		neg		neg		175-580	
	[%]	[%]	(neg/pos)	(neg/pos)	(neg/pos)	(neg/pos)	[G/l]	[G/l]
POBA-1	3	2	pos	neg	neg	neg	196	292
POBA-2	8	1	pos	neg	neg	neg	316	291
POBA-3	2	2	neg	neg	neg	neg	276	410
Mean ± SD	4 ± 3	2 ± 1	n.a.	n.a.	n.a.	n.a.	263 ± 61	331 ± 68
3.5-1	3	4	pos	neg	neg	neg	329	443
3.5-2	3	7	pos	neg	neg	neg	230	407
3.5-3	1	4	neg	neg	neg	neg	160	431
Mean ± SD	2 ± 1	5 ± 2	n.a.	n.a.	n.a.	n.a.	240 ± 85	427 ± 18
1x6-1	3	1	pos	neg	neg	neg	172	285
1x6-2	1	1	pos	neg	neg	neg	167	317
1x6-3	5	3	pos	neg	neg	neg	323	411
Mean ± SD	3 ± 2	2 ± 1	n.a.	n.a.	n.a.	n.a.	221 ± 89	338 ± 65
3x6-1	2	2	pos	neg	neg	neg	303	353
3x6-2	7	3	pos	neg	neg	neg	310	453
3x6-3	3	3	pos	neg	neg	neg	240	344
Mean ± SD	4 ± 3	3 ± 1	n.a.	n.a.	n.a.	n.a.	284 ± 39	383 ± 61

Summary of blood parameters before intervention (Pre int.) and at 4-week follow-up (4-week FU). Some pigs showed hypochromia and hemoglobin values around or slightly below estimated normal values for pigs upon arrival from the breeder.
